# Climate change effects on mental health: are there workplace implications?

**DOI:** 10.1093/occmed/kqac100

**Published:** 2022-09-28

**Authors:** S K Brooks, N Greenberg

**Affiliations:** Department of Psychological Medicine, King’s College London, London SE5 9RJ, UK; Department of Psychological Medicine, King’s College London, London SE5 9RJ, UK

## Abstract

**Background:**

Climate change can negatively affect mental health, and poor mental health can negatively affect work. However, less is known about the relationship between mental health and workplace behaviours within the climate change context.

**Aims:**

To explore existing literature relating to climate-induced mental ill-health as a potential predictor of workplace behaviours.

**Methods:**

Scoping review, searching five databases for relevant literature using two separate search strategies.

**Results:**

Only five studies with any relevant data were found. Results could not be easily synthesized because each of the five considered different work-related outcomes. However, the available data suggest that the psychological impact of extreme events could lead to increased job tension, higher turnover intentions and workplace hostility. Stress about extreme weather could also impede the ability to make essential work-related decisions and, for those who work in the environmental sector, concerns about climate could lead to overcommitment to work. There was some evidence that social support might lessen the effects of climate-induced stress on work outcomes.

**Conclusions:**

Very little literature considers the impacts of climate change on employees’ mental health and associated workplace function. The available evidence suggests there are potential negative impacts which may be mitigated by social support. It is important for future research to explore ways of supporting staff and fostering resilience.

## Teaser

Despite recent academic interest in how climate change can affect mental health and how mental health can affect workplace behaviours, the three topics are rarely considered together. Little is known about how the effects of climate change on workers’ mental health might subsequently impact workplace function. This scoping review found very few studies considering ‘climate-induced mental ill-health’ as a potential predictor of work outcomes and calls for more research to fill this gap in understanding.

**Key learning points**

**What is already known about this subject:**

- Climate change has been associated with negative mental health effects ranging from increased stress to psychological disorders such as anxiety and depression.
- Poor mental health can negatively affect workplace behaviours including productivity, absenteeism, presenteeism and turnover intentions.

**What this study adds:**

- Our scoping review revealed that the three concepts (mental health, climate change and workplace behaviours) are rarely discussed together.
- To date, there is very sparse literature on whether, and how, the mental health effects of climate change subsequently affect workplace behaviours.
- The available data suggest that climate-induced mental ill-health could have negative impacts on the workplace if employees are not appropriately supported.

**What impact this may have on practice or policy:**

- There is an urgent need for research addressing the relationship between mental health and workplace behaviour in the context of climate change.
- The available evidence suggests that workplaces should prepare to mitigate potential negative impacts on mental health and consequentially on functioning, by ensuring high levels of social support at work.

## Introduction

Recent years have seen substantial meteorological changes including record temperatures and a rise in heatwaves, droughts and bushfires, as well as accelerated glacier retreat increasing the risk of flooding. Climate change has also increased the frequency and severity of extreme events such as hurricanes and tropical storms [1]. Understandably, the potential health impacts of climate change have been the subject of much scholarly research, but climate change’s effects on mental health are less frequently explored: an umbrella review of 94 systematic reviews on the health impacts of climate change [2] found that only 10/94 considered mental health outcomes, with most focusing instead on infectious diseases or mortality.

Much of the research which does explore the impact of climate change on mental health focuses on the immediate, acute effects of exposure to extreme events such as natural disasters. Evidence suggests that experiencing a climate-related disaster can increase risk of post-traumatic stress disorder (PTSD), anxiety, distress, depression and substance/alcohol abuse [2,3]. More gradual and prolonged effects of climate change, such as temperature increases and subsequent heatwaves, can also impact on mental health: meteorological changes can lead to mental health effects ranging from more minimal impacts on stress to diagnoses of clinical disorders [3] and have been associated with mental health-related hospital admissions, suicide, exacerbation of pre-existing mental health conditions, sleep problems and fatigue [2]. These effects could be a direct result of consequences of gradual climate change such as negatively affected livelihood opportunities, economies, food insecurities, migrations and forced displacements [4]. There is also an emerging literature on the concept of ‘eco-anxiety’—that is, distress and anxiety about the future, caused by the frequent reports of an impending climate crisis, which can affect any individuals with concerns about climate change, not just those who have been personally affected [5]. Anxiety around climate change appears to be on a spectrum, encompassing worry about threatened livelihoods, worry for future generations and worry for an ‘apocalyptic’ future, which can lead to feelings of hopelessness and disempowerment [6].

Numerous occupational groups may be substantially affected by climate change. For example, in extreme climate-related events, responders—such as disaster relief workers and emergency services—are at risk of poor psychological outcomes. These staff’s post-disaster mental health may be worsened by various factors such as injury; exposure to traumatic scenes; and more severe impact of the disaster on personal and professional life; and moderated by social support and managerial support [7]. Research has also identified various occupational groups as being particularly vulnerable to the effects of gradual climate change, in particular outdoor workers such as those in the agricultural, construction and manufacturing sectors [1,8,9].

If employees who have been involved in extreme climate-related events or affected by meteorological changes are struggling with their mental health, what does this mean for their work? Existing (non-climate-related) research suggests that mental ill-health can negatively affect several workplace behaviours, including productivity, absenteeism and presenteeism [10]. There is also evidence from healthcare research that working in crisis situations can lead to burnout which in turn can negatively affect turnover intentions [11] and mental ill-health, such as depression, can affect willingness to work [12]. Kjellstrom *et al.*’s [13] framework for conceptualizing the relationship between heat stress, health and work/socioeconomic effects suggests that heat stress can lead to psychological changes which can lead to diminished performance capacity, increased accident risk, reduced work capacity, occupational injuries and reduced economic output. However, there appears to be little empirical evidence exploring the relationship between climate-affected mental health and workplace behaviours. A scoping review was therefore carried out to ascertain whether this has been adequately explored in academic literature.

## Methods

Following Arksey and O’Malley’s guidelines for scoping reviews [14], we identified the following research question: *how might mental health consequences of climate change alter work-related behaviours?* A search strategy was designed which consisted of (i) terms relating to climate-related mental health, such as ‘eco-anxiety’ and the term ‘climate’ within two words of the term ‘anxiety’; (ii) terms relating to workplace behaviours; and (iii) terms relating to employees. Due to lack of relevant findings from this search, a second search strategy combining terms related to (i) climate change, (ii) mental health, (iii) workplace behaviour and (iv) employees was developed (see Supplementary Appendix 1, available as Supplementary data at *Occupational Medicine* Online, for both strategies). Five databases were searched in June 2022: Embase, Global Health, Medline, PsycInfo and Web of Science. To be included, studies needed to present primary data, published in peer-reviewed journals, relating to the impact of climate change on mental health and how this in turn affects work outcomes. ‘Mental health’ included both general well-being and clinical disorders such as depression and PTSD, and relevant work outcomes included absenteeism, presenteeism, productivity, turnover and other work-related outcomes such as organizational citizenship. ‘Climate change’ referred to any meteorological changes including both gradual changes such as increased temperatures and extreme weather events such as floods and earthquakes. Earthquakes were included as there is increasing evidence that climate change may potentially be indirectly associated with earthquake frequency: for example, it has been suggested that tectonic plate movement may be associated with the melting of glaciers [15,16] and rising soil temperatures [16]. Citations were imported into EndNote where one author reviewed titles, abstracts and then full texts against the inclusion criteria. Papers which underwent full-text screening were discussed with the second author. Thematic analysis [17] was used to explore whether there were any themes emerging from the included studies.

## Results

The first search yielded 329 studies, of which 12 were duplicates. Title screening resulted in 283 exclusions and abstract screening resulted in a further 17. Nine remaining full texts were screened but only one study had any relevance to the review. The second search yielded 495 studies, of which five were duplicates, and 390 were excluded based on title. A further 45 were excluded based on abstract leaving 55 for full-text screening; of these, four contained some data relevant to the research question. Combined with the study found in the first search, this left five studies for inclusion. Characteristics of these studies are summarized in Table 1. Two described quantitative data [18,19] and three described qualitative [20–22]; studies were from New Zealand (*n* = 3), Australia (*n* = 1) and the USA (*n* = 1). Most focused on single events: earthquakes (*n* = 2) and hurricanes (*n* = 1). Another explored the effect of extreme weather such as droughts, and finally one considered anxiety around climate change in general. Occupational groups included teachers (*n* = 1), environmental workers (*n* = 1), dairy farmers (*n* = 1), employees of human services organizations (*n* = 1) and employees from multiple occupational groups (*n* = 1).

**Table 1.** Characteristics of relevant studies

Study (authors, year)	Country	Occupational group	Climate event	Participants	Measures
Hochwarter et al. (2008) [18]	USA	Various	Hurricanes	Study 1: 368 blue-collar or white-collar workers; Study 2: 167 employees of a manufacturing organization; Study 3: 120 employees of a refuse disposal company; Study 4: 247 blue-collar or white-collar workers; Study 5: 120 employees of a municipal agency	Positive and negative affect; hurricane-induced job stress; perceived resources; job tension; job satisfaction; work intensity
Kuntz et al. (2013) [19]	New Zealand	Teachers	Earthquake	125 teachers of primary, secondary or intermediate schools	Personal disaster impact; school disaster impact; perceived school disaster responsiveness; role conflict; role overload; burnout; turnover intentions
Noy et al. (2022) [20]	Australia	Environmental workers	Climate change in general	Eight senior managers (interviews) and nine employees (two focus groups)	Interviews and focus groups exploring strengths, challenges and supports for mental health and well-being
Tisch and Galbreath (2018) [21]	New Zealand	Dairy farmers	Extreme weather events from ­climate change, e.g. drought	38 farm owners and general managers	Interviews exploring the nature and dynamics of climate change impacts
Van Heugten (2018) [22]	New Zealand	Employees of human services organizations	Earthquake	28 frontline workers and 15 managers	Interviews exploring challenges and opportunities experienced in the workplace in the aftermath of a disaster

The two quantitative studies examined different variables: Hochwarter *et al.* [18] found that ‘hurricane-induced job stress’ predicted job tension but having high levels of resources (such as personal coping abilities and workplace support) neutralized the adverse effects of hurricane-induced stress and also led to increased job satisfaction even as hurricane-induced stress increased. For those with low resources, hurricane-induced stress increased job tension and decreased job satisfaction and work effort. Kuntz *et al.* [19] found that, in a sample of teachers who had experienced an earthquake, emotional exhaustion mediated the relationship between role overload and turnover intentions, where high role overload was related to emotional exhaustion which in turn was related to higher turnover intentions.

Qualitative data also focused on different work-related outcomes, namely workplace hostility; overcommitment to work; and work-related decision-making. A study focusing on employees from human services organizations who had experienced earthquakes [22] found that stress was perceived to have been indirectly affected by the earthquake (i.e. the earthquake had led to lack of basic resources and office space, which was perceived to have caused stress) and this stress was subsequently perceived to have led to emotional outbursts at work, incivility in the workplace, aggression and bullying.

In Noy *et al.*’s [20] study of environmental workers, participants reported a sense of urgency and anxiety about climate change which they perceived to have led to overcommitment: together with insufficient resources, this meant they worked long hours and struggled with work–life balance. Some reported a sense of isolation due to their friends and families not wanting to confront environmental issues, and this isolation again contributed to overwork.

Finally, in Tisch and Galbreath’s [21] study, prompt decision-making was seen as essential for farmers (as those who did not make prompt decisions were perceived to suffer more badly in extreme weather events such as droughts) and workplace decision-making was perceived to be hampered by stress about extreme weather.

## Discussion

The key finding from this review was the paucity of research focusing on how climate-induced mental ill-health can impact on workplace behaviours. We found some evidence that the impact of extreme events on mental health may be associated with job tension, turnover intentions, workplace hostility, difficulties making work-related decisions and overcommitment to (environment-related) work, as well as some evidence that social support might mitigate the effects of climate-related mental ill-health on work.

It is well-established that there are several ways in which the lives and mental health of various occupational groups may be impacted by climate change. Those who respond to extreme events or whose organizations are affected by extreme events by necessity or chance may experience traumatic exposure to death, injury and destruction; fear for one’s own life; illness or injury, or knowing people (e.g. family, colleagues) who were injured or killed in the event; major changes to job role in order to respond to the crisis; loss of homes; loss of employment; negative media coverage or response from the public relating to their work responding to the crisis; and disruption to infrastructure, transport and medical care [7]. The more gradual, prolonged effects of climate change can also directly affect employees’ lives, for example causing them to lose significant work hours due to extreme temperatures, which can lead to severe economic consequences for those affected [1]. These impacts on personal and professional lives can be profound and may have far-reaching and long-lasting effects on mental health. What is not yet fully understood is how these effects on mental health may impact on the workplace; however, the limited data available suggest that they could lead to negative work behaviours such as tension, hostility, difficulty making decisions, intentions to leave and overworking. Additionally, the mental health impacts of climate change may have knock-on effects even for employees whose mental health is not directly impacted: for example, after extreme weather events such as flooding, organizations tend to see a great deal of absenteeism with not enough staff to perform normal delivery of services [23]—this may subsequently lead to work overload for those who do go in, which could affect their mental health and workplace behaviours.

It should also be noted that extreme weather events can have some positive psychological outcomes, as well as negative: for example, employees involved in the relief and recovery efforts after natural disasters frequently sometimes post-traumatic growth [7], for example finding new meaning in life, feeling a sense of purpose or renewed appreciation for life. It is possible that these could in turn lead to positive work-related outcomes, such as increased job satisfaction and increased appreciation for, or strengthened relationships with, colleagues. However, despite searching for ‘post-traumatic growth’ as a keyword, our searches did not uncover any studies explicitly examining the relationship between climate-related post-traumatic growth and work-related outcomes.

To our knowledge, this is the first review to summarize findings related to how climate-affected mental health might affect the workplace. However, it is important to note that there were very few relevant studies found, and none which considered the same variables: the included studies are heterogeneous in terms of the occupational groups studied, the methods and measures used and the outcomes examined, which makes it very challenging to generalize from the findings.

The main limitation of the review is the lack of relevant literature found in the searches, which appears to reflect a gap in peer-reviewed academic literature relating to climate change effects. The use of two entirely separate search strategies, neither of which found a great deal of relevant literature, supports this view. However, it is also possible that the searches missed out on relevant papers, perhaps by not being broad enough or not searching enough databases: less specific search strategies carried out across a larger number of databases might yield more relevant results. Additionally, searching grey literature may also yield relevant data. Another limitation of the review process is the fact that one author carried out all screening; ideally, double screening by a second author would strengthen the reliability of the screening process.

It is possible that there are other studies, not located in our searches, which do consider the workplace implications of climate-related mental ill-health: if this was only discussed briefly or peripherally in a paper with a focus on different outcomes, the search strategy may not have uncovered them. However, whilst this may be the case, we suggest that this review shows a clear lack of studies with a focus specifically on the impact of climate-related mental health on work. Additionally, it must be noted that three of the included studies involved extreme events, rather than gradual environmental changes; the effects of extreme events on mental health may be different to the effects of gradual prolonged climate change. More research is needed which explores mental health and work-related effects of gradual changes in the environment rather than discrete extreme events.

We suggest that the relationship between climate-induced mental ill-health and work outcomes, as a broad topic, urgently requires more academic attention. The paucity of relevant studies found in this review illustrates how little empirical evidence currently exists on this topic. There are also specific areas within this broad topic which could benefit from research. For example, the impact of climate on mental health on workers responding to climate-related disasters may be a particularly pressing area. From our screening process in this review, it is also clear that more research on the mental health and work-related impacts of slowly evolving environmental changes (as opposed to singular extreme events) is also needed as this may impact on all workers. Perhaps most importantly, research addressing how to reduce climate-related mental ill-health (and its subsequent effects on workplace behaviours) is also needed: we did not find any studies considering workplace interventions. In fact, it is suggested that workplaces struggle to support those with eco-anxiety and that employees perceive that mental health practitioners have limited understanding of eco-anxiety as a normal response to a real threat [18]. Further research is needed to understand how best to support employees affected by climate change. It has been suggested that occupational health providers need to have the ability to recognize and mitigate climate change-related health effects, including mental health effects [24] but further research on this topic is needed to provide appropriate guidance for providers.

Overall, based on what is already known about how workplaces can support the (non-climate-related) mental health of employees [25], we suggest that in the meantime organizations—and their workers—can benefit from adequate training to cope with extreme events; support from managers; support from colleagues; and education regarding effective coping strategies. However, climate change is likely to continue impacting mental health as temperatures continue to rise and extreme events become more frequent [26] and so it is becoming increasingly pressing those employers (particularly within organizations who are likely to be affected by climate change and those in at-risk professions) should be increasingly focused on supporting their employees’ mental health.

## Supplementary Material

kqac100_suppl_Supplementary_Material
