# Supplementary material for: Climate change effects on mental health: are there workplace implications?
Source: Occup Med (Lond). 2022 Sep 28;73(3):133–7. doi: 10.1093/occmed/kqac100 (PMC10132205; doi:10.1093/occmed/kqac100)
Supplement: kqac100_suppl_Supplementary_Material [file kqac100_suppl_supplementary_material.docx]

**Supplementary Appendix 1: Search strategies**

**Search strategy 1**

(climate NEAR anxiety) or (climate NEAR risk perception*) or (climate NEAR fear) or eco-anxiety or eco-depression or eco-anger or climate-induced psychological or climate-induced mental or (climate NEAR concern) or (climate NEAR panic) or (climate NEAR distress) or ("global warming" NEAR fear*) or ("global warming" NEAR risk perception*) or ("global warming" NEAR concern) or ("global warming" NEAR anxiety) or ("global warming" NEAR distress) or ("global warming" NEAR panic) or (climate NEAR worry) or (climate NEAR worries) or ("global warming" NEAR worry) or ("global warming" NEAR worries)

AND

work behav* or workplace behav* or "job performance" or "work performance" or absenteeism or presenteeism or turnover or organisational climate or organizational climate or organisational citizenship or organizational citizenship or organisational behav* or organizational behav* or occupational stress or work stress or workplace stress or productivity

AND

employee* or worker* or staff or personnel

**Search strategy 2**

(wellbeing or well-being or anxiety or panic or "post-traumatic stress" or PTSD or distress or "mental health" or depression or neurosis or burnout or resilien* or "post-traumatic growth" or psychological) AND (work behav* or workplace behav* or "job performance" or "work performance" or absenteeism or presenteeism or turnover or organisational climate or organizational climate or organisational citizenship or organizational citizenship or organisational behav* or organizational behav* or occupational stress or work stress or workplace stress or productivity) AND ("climate change" or "climate risk" or "extreme weather" or drought or "extreme heat" or "extreme temperature" or flood* or "climate hazard*" or cyclone* or wildfire* or heat-wave* or heat wave* or heatwave* or hurricane* or "forest fire*" or storm* or "weather-related disaster*" or tornado* or typhoon* or earthquake* or hurricane* or tsunami* or "global warming") AND (employee* or worker* or staff or personnel)
